# Supplementary material for: Tissue-Specific Regulation of HNK-1 Biosynthesis by Bisecting GlcNAc
Source: Molecules. 2021 Aug 26;26(17):5176. doi: 10.3390/molecules26175176 (PMC8434142; doi:10.3390/molecules26175176)
Supplement: Supplementary file 1 [file molecules-26-05176-s001.zip › molecules-1341288-supplementary.pdf]

Supplementary Material

Table S1. MM/GBSA energies of *N*-Glycan binding to GlcAT-P and GlcAT-S. Sum of ΔE_{VDW} (Van der Walls energy), ΔE_{ELE} (electrostatic) contribution to molecular mechanics energy, and ΔE_{GB} (polar) and ΔE_{NP} (non-polar) contribution to solvation free energy sums up to total binding energy (ΔE_{TOTAL}) All the energies ± standard deviations are in kcal.mol^{-1}.

**Structure**	**Glycan**	**branch**	**ΔE_{VDW}**	**ΔE_{ELE}**	**ΔE_{GB}**	**ΔE_{NP}**	**ΔE_{TOTAL}**
GlcAT-P	GGnGGnbi	α(1,3)	–62.8±9.4	–83.6±20.0	103.7±19.2	–5.8±0.8	–48.6±8.2
	Bisect-GGnGGnbi	α(1,3)	–62.0±9.0	–149.5±22.9	157.7±18.8	–6.9±0.9	– 60.7±11.3
	GGnGGnbi	α(1,6)	–53.3±8.7	–104.1±25.5	123.5±22.9	–5.6±0.8	–39.5±8.9
	Bisect-GGnGGnbi	α(1,6)	–36.5±11.5	–44.4±23.3	63.4±25.6	–3.7±1.1	–21.1±8.8
GlcAT-S	GGnGGnbi	α(1,3)	–44.3±9.0	–105.1±17.3	110.3±17.3	–4.8±0.9	–44.0±7.3
	Bisect-GGnGGnbi	α(1,3)	–54.6±7.0	–78.2±17.4	92.3±17.0	–4.9±0.7	–45.3±7.5
	GGnGGnbi	α(1,6)	–66.8±11.2	–71.5±21.4	94.2±19.6	–6.2±0.9	–50.2±9.4
	Bisect-GGnGGnbi	α(1,6)	–69.3±9.0	–128.1±41.9	147.6±43.8	–6.6±1.0	–56.4±9.0
